# Evidence That Non-Syndromic Familial Tall Stature Has an Oligogenic Origin Including Ciliary Genes

**DOI:** 10.3389/fendo.2021.660731

**Published:** 2021-06-04

**Authors:** Birgit Weiss, Birgit Eberle, Ralph Roeth, Christiaan de Bruin, Julian C. Lui, Nagarajan Paramasivam, Katrin Hinderhofer, Hermine A. van Duyvenvoorde, Jeffrey Baron, Jan M. Wit, Gudrun A. Rappold

**Affiliations:** ^1^ Department of Human Molecular Genetics, Institute of Human Genetics, Ruprecht Karls University Heidelberg, Heidelberg, Germany; ^2^ Department of Pediatrics, Leiden University Medical Center, Leiden, Netherlands; ^3^ Section on Growth and Development, National Institute of Health, Bethesda, MD, United States; ^4^ Computational Oncology Group, Molecular Diagnostics Program at the National Center for Tumor Diseases (NCT) and German Cancer Research Center (DKFZ), Heidelberg, Germany; ^5^ Institute of Human Genetics, Ruprecht Karls University Heidelberg, Heidelberg, Germany; ^6^ Department of Clinical Genetics, Leiden University Medical Center, Leiden, Netherlands

**Keywords:** oligogenic inheritance, non-syndromic tall stature, growth, ciliary genes, growth plate, exome sequencing

## Abstract

Human growth is a complex trait. A considerable number of gene defects have been shown to cause short stature, but there are only few examples of genetic causes of non-syndromic tall stature. Besides rare variants with large effects and common risk alleles with small effect size, oligogenic effects may contribute to this phenotype. Exome sequencing was carried out in a tall male (height 3.5 SDS) and his parents. Filtered damaging variants with high CADD scores were validated by Sanger sequencing in the trio and three other affected and one unaffected family members. Network analysis was carried out to assess links between the candidate genes, and the transcriptome of murine growth plate was analyzed by microarray as well as RNA Seq. Heterozygous gene variants in *CEP104, CROCC, NEK1, TOM1L2*, and *TSTD2* predicted as damaging were found to be shared between the four tall family members. Three of the five genes (*CEP104, CROCC*, and *NEK1*) belong to the ciliary gene family. All genes are expressed in mouse growth plate. Pathway and network analyses indicated close functional connections. Together, these data expand the spectrum of genes with a role in linear growth and tall stature phenotypes.

## Introduction

Human height is a highly polygenic complex trait, largely determined by genetics. Most of our understanding of the genetic architecture of height has derived from children with abnormal growth patterns and single gene defects (rare variants of large effect) (1, 2). At the other end of the spectrum, polygenic risk, measured as the sum of thousands of common risk alleles with small effects also contributes to height (3). Oligogenic effects fall between these two extremes, which—so far—have not been well characterized. Evidence has accumulated over the last few years that clinical outcomes can be influenced or modulated by the concerted action of small numbers of rare gene variants, giving rise to oligogenic traits.

Normal growth requires signaling through many different pathways at the growth plate. Multiple hormones, growth factors, and cytokines as well as paracrine factors regulate growth plate function and thereby linear growth. We have studied the genetic profile of a family with a son with extremely tall stature, a very tall father and normal statured mother by exome sequencing and extended the analysis to other family members. Tall stature is defined as height of more than two standard deviations (SDs) above the mean for a defined population (www.icped.org). In families with multiple affected individuals, genetic top hits can be compared between individuals sharing the same phenotype and those who are unaffected, thereby subtracting the common genetic background. If multiple gene variants are identified that may contribute to a specific phenotype, it can be of interest to determine how these genes interact in networks. Connected genes within a pathway can provide useful insights on the biology and function. Lastly, expression analysis in growth plates may provide further indications on functionality.

## Method

### Growth Analysis

Birth weight and length were expressed as standard deviation score (SDS) for Swedish references (4). Height and head circumference of the index case and his sister were expressed as SDS for the 1997 Dutch nationwide reference (5). Height SDS of the parents and their siblings was calculated based on the 1965 Dutch nationwide reference (6). Reported height in young adulthood of the paternal grandparents and the grandfather’s sister was expressed as SDS taking into account secular trend (“adjusted height SDS”) (7).

Sitting height, leg length, and sitting height/height ratio were expressed as SDS for age and sex based on the 1997 Dutch reference (8). Arm span and arm span/height ratio were expressed as SDS for Dutch children (9, 10). Weight-for-height and body mass index were expressed as SDS based on the 1980 nationwide growth study (11).

To analyze growth data in a multigenerational family is a challenge because of the downward course of height in anybody’s lifetime (due to shrinking) and the phenomenon of secular trend in the mean height of a given population that has been prominent in most countries in the past century. Therefore, for a proper analysis of height in a multigenerational family, it is necessary to obtain information about the approximate age at which the height measurement was taken so that the height in young adulthood can be estimated, followed by a comparison with the reference data in the year that the person was 21 years of age. Fortunately, good longitudinal data on shrinking by age have been published, and for the Netherlands detailed information is available about mean (SD) height of the population over the last century (7). This enabled us to estimate height SDS adjusted for shrinking and secular trend.

### Ethical Approval

This study was part of the research protocol (P06.118) “Genetic diagnosis of very tall stature”, Leiden University Medical Center. Written informed consent was received from all participants prior to their inclusion in the study. The study was also part of the Research Program “Whole exome genetic analyses in rare hereditary diseases” of Heidelberg University. The study was conducted in accordance with the guidelines of the WMA Declaration of Helsinki and the Department of Health and Human Services Belmont Report.

### Exome Sequencing and Filtering

Agilent SureSelect Human All Exon V4 was used to capture the exons. Sequencing was done using the Illumina HiSeq 2000 system. Raw sequence data was mapped to the 1,000 genomes phase II reference genome (GRCh37 version hs37d5) using BWA 0.6.2, and duplicates were removed using PICARD. The three samples (parents and index case) showed an average coverage of 176×, and >99% of bases in the autosomal chromosomes of the target regions have >=10× coverage and >=20 QUAL score (**Supplementary Figure 1**) The SNVs and indels were called along with parent samples using Platypus (version 0.8.1.1). In-house pipelines were used to annotate the variants with dbSNP, 1,000 genomes, gnomAD and local control databases. ANNOVAR and gencode (version 19) were used for the functional annotation of the variants. For further analysis, SNVs and indels with a read depth of at least 10× and minimum QUAL score of 20 were considered. Non-synonymous (missense, stop gain, stop loss) and splice site affecting SNVs as well as all exonic indels were filtered further. The genotype predicted by Platypus was used to assign genotypes to the variants. The Minor allele frequency (MAF) from gnomAD (version 0.2.1) genes and exomes was used to define the variant’s rareness in the population. Variants with MAF >0.1% were removed. Furthermore, a set of 1,198 exomes and 3,910 WGS samples from the in-house database was used as control to remove common variants and sequencing artefacts. Variants present in more than 1% of the control samples were removed.

The functional effect of the remaining variants was predicted using four *in silico* prediction tools: PolyPhen2, PROVEAN, SIFT and MutationTaster. Variants predicted with a CADD score above 20 and as damaging by at least two of the four *in silico* prediction tools were used for further analysis. A CADD score above 20 indicates the variant to be within the 1% most deleterious substitutions in the human genome.

### Sanger Sequencing of Variants

Twenty-eight variants were confirmed by Sanger sequencing. Polymerase chain reaction (PCR) with primers indicated in **Supplementary Table 1** was performed with HotStar Taq Polymerase and products analyzed on agarose gels and subsequently sequenced using an ABI machine (Genewiz).

### Network and Pathway Analysis by Ingenuity Software

QIAGEN IngenuityPathway Analysis (IPA; https://digitalinsights.qiagen.com/) was applied to predict functional connections, and their interpretation was carried out in the context of protein networks that comprise protein–protein interactions and related biological functions, and canonical signaling pathways.

### Databases

TGP (https://browser.1000genomes.org); GnomAD (https://gnomad.broadinstitute.org/); CADD score (https://cadd.gs.washington.edu); dbSNP (https://www.ncbi.nlm.nih.gov); GTEx database **(** www.gtexportal.org); PROVEAN/SIFT (http://provean.jcvi.org/index.php); Polyphen2 (http://genetics.bwh.harvard.edu/pph2/); Mutation Taster (http://www.mutationtaster.org/); GWAS (www.ebi.ac.uk); IPA Ingenuity Systems (https://digitalinsights.qiagen.com/).

### Expression Analysis in Growth Plates

Gene expression analysis in mouse growth plate was carried out to analyze (1) *growth plate specificity*: comparing mRNA expression in growth plate of 1-week-old mice with expression in three different soft tissues (lung, kidney and heart) using microarray (12). (2) *spatial regulation*: comparing mRNA expression in proliferative and hypertrophic zones in the 1-week-old mouse growth plate by RNA-Seq (13); and (3) *temporal regulation*: comparing mRNA expression at 1 and 4 weeks of age in proliferative and hypertrophic zones of mouse growth plate by RNA-Seq (13).

## Results

### Clinical Data

**Figure 1:**
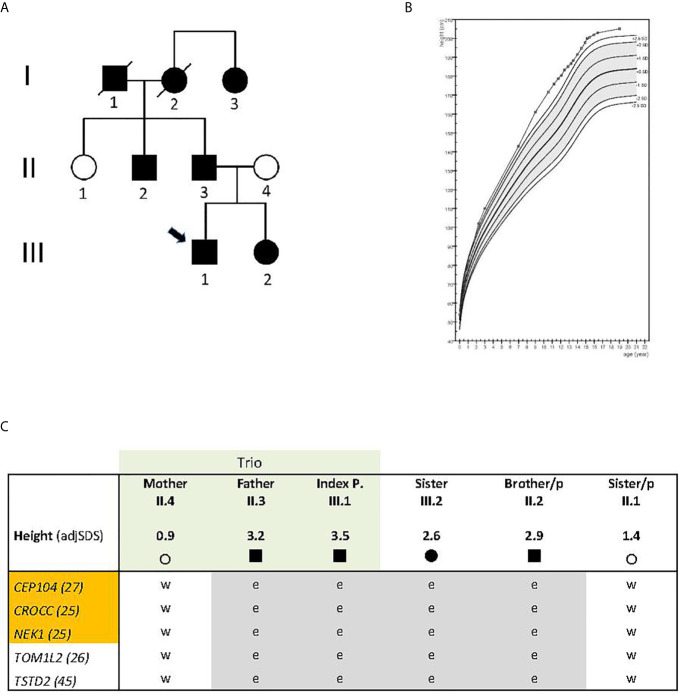
**(A)** Pedigree with non-syndromic familial tall stature. Arrow indicates index individual; circle, females, square, males; **(B)** Growth curve of the index case, plotted on the reference data from the Dutch 4^th^ nation-wide growth study (8). Epiphysiodesis was performed at 15.1 years. Layout prepared using Growth analyser.org; **(C)** Gene variants shared between the four tall family members and not present in family members with normal height (in gray color). Exome sequenced trio is indicated in light green; orange color indicates ciliary genes. A nonsense variant was identified in *TSTD2;* the others are missense variants. CADD scores are given in numbers behind the gene symbol; adjSDS, stands for standard deviation score adjusted for secular trend in the population;/p, indicates paternal side; circle, female, square, male; e, heterozygous; w, wild type.

The index case (**Figure 1A**, individual III.1) was born as the first child of healthy parents of Dutch origin. His father’s (individual II.3) height at 18 years was 199.8 cm (3.2 SDS), and his mother’s (individual II.4) height was 172 cm (0.9 SDS); his sister (III.2) who was two years younger than the proband reached an adult height of 187 cm (2.6 SDS). The father´s brother’s (II.2) height was 198 cm (2.9 SDS), and his sister´s height (II.1) 176 cm (1.4 SDS). Reported height of the (deceased) paternal grandfather (I.1) in young adulthood was 193 cm (2.7 SDS adjusted for secular trend), of the (deceased) paternal grandmother (I.2) 176 cm (2.2 SDS), and her 6 years younger sister (I.3) also 176 cm (2.1 SDS) (**Figure 1A**).

At the age of 10.5 years, the proband was referred to the pediatric endocrine clinic because of tall stature. Previous growth data included a birth weight of 3.8 kg (0.5 SDS) and length of 54 cm (1.8 SDS). During the first year, length increased to 2.0 SDS, weight-for-age remained close to 2.0 SDS, and head circumference decreased from 2.2 to 1.3 SDS. During childhood, his height gradually increased to 3.5 SDS. At age 10.5, his height was 172 cm (3.8 SDS), sitting height 89.5 cm (4.1 SDS), leg length 82.3 cm (4.3 SDS), sitting height/height ratio 0.5203 (0.2 SDS), arm span 169.5 cm (3.7 SDS), arm span/height ratio 0.7 SDS, head circumference 56 cm (1.5 SDS), and weight 60.0 kg (BMI 1.5 SDS). Shoe size was 43. He had no hyperlaxity or arachnodactyly, and established causes of tall stature (Klinefelter syndrome, pituitary gigantism, hyperthyroidism, and genetic aberrations of the CNP-NPR2 axis) were excluded by karyotype, serum (normal IGF-I, IGFBP-3, FSH, LH, testosterone, FT4 and TSH) and candidate gene analysis (*NPPB*, *NPPC*, *NPR2* and *NPR3*). A duplication of the SHOX gene region was excluded by MLPA (MRC Holland). At follow-up, full pubertal development (testes ˃16 ml) was reached at 14.4 years, and at 14.9 years, height was 197.8 cm (3.0 SDS) and predicted adult height 208.6 cm (3.5 SDS). Epiphysiodesis was performed at 15.1 years of age and at 16.7 years his adult height was 202.8 cm (3.0 SDS). His growth curve is shown in **Figure 1B**.

The boy’s sister was born with a weight of 3.6 kg (0.4 SDS) and a length of 52 cm (1.2 SDS). During the first year, her length increased to 2.5 SDS, weight remained at 1.3 SDS and the head circumference was −0.5 SDS. During childhood, her height SDS was approximately 2.2 SDS. At 13.2 years of age, height was 177.2 cm (2.3 SDS), sitting height 88.4 cm (1.2 SDS), sitting height/height ratio −1.2 SDS, arm span 180.8 cm (2.7 SDS), arm span/height ratio 1.1 SDS, weight 64.0 kg (BMI 0.7 SDS), pubertal stages B5, P3, A3, blood pressure 118/61 mm Hg. Menarche occurred at 14.0 years. Adult height was 187 cm (2.6 SDS) (**Figure 1C**).

### Identification of Genetic Candidate Variants for Tall Stature

**Figure 2:**
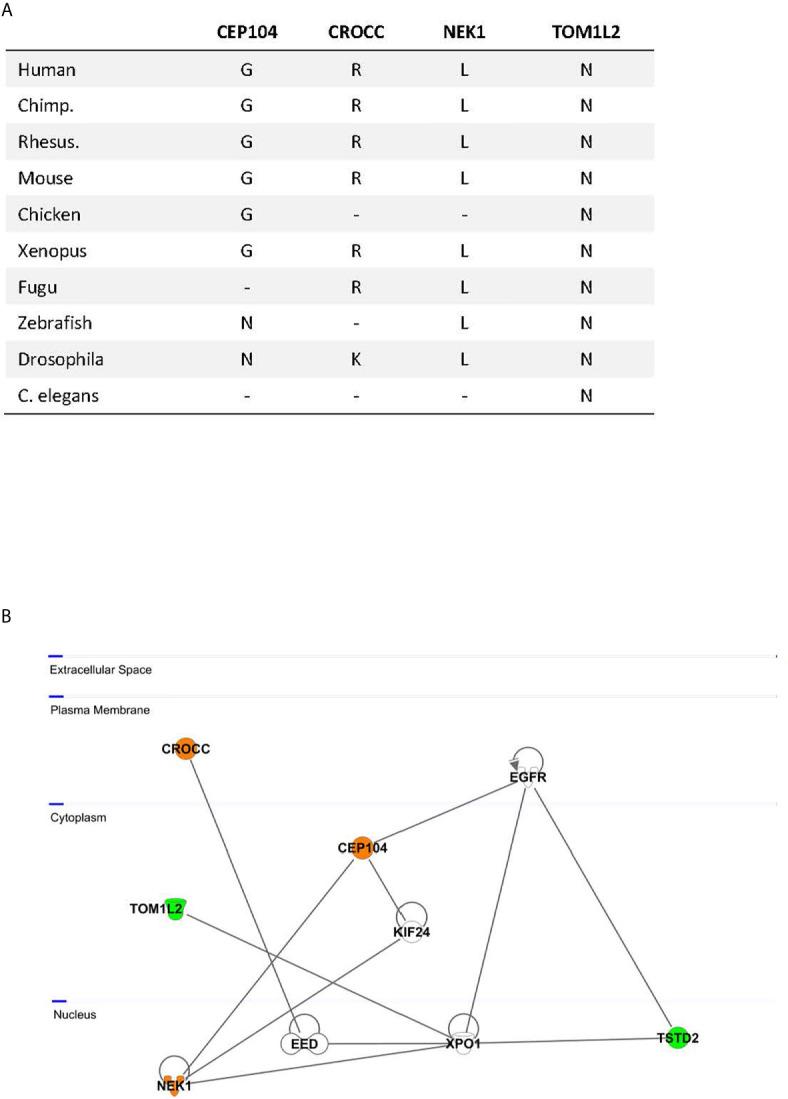
**(A)** Evolutionary conservation of the affected amino acids by missense mutations; −, no ortholog; a nonsense variant was identified in *TSTD2*; **(B)** Network Analysis by Ingenuity pathway analysis. Identified candidate gene products are highlighted in color; orange, proteins encoded by ciliary genes; green, proteins encoded by non-ciliary genes.

Exome sequencing was carried out in the index case (adjusted adult height 3.5 SDS), his father (height 3.2 SDS), and his mother (height 0.9 SDS) (**Figure 1A**, individuals III.1, II.3, II.4 respectively). After bioinformatics filtering, all rare, predicted damaging variants found to be shared between the affected father and his son but not present in the mother with normal height, with a CADD score above 20 and not rated as polymorphisms in gnomAD, were considered (**Supplementary Table 2A**). Twenty-eight selected rare gene variants were followed up and analyzed in three closely related family members by Sanger sequencing: two individuals with tall stature (II.2 and III.2) and one with normal height (II.1) (**Figure 1A**). (Data of deceased grand-parents, not shown). Four family members with tall stature shared identical heterozygous variants, which were not present in the family members with normal stature, in the following genes: Centrosomal Protein 104 (*CEP104)*, Rootletin *(CROCC)*, Serine/threonine-protein kinase *(NEK1)*, Thiosulfate Sulfurtransferase-like Domain containing 2 (*TSTD2)* and Target Of Myb1-Like 2 *(TOM1L2)*, suggesting that these were potentially relevant for the phenotype (**Figure 1C**; **Table 1** **)**. All identified missense variants were predicted to be damaging or probably damaging (**Supplementary Table 2**) and led to changes in highly evolutionary conserved amino acids **(** **Figure 2A** **)**; in *TSTD2*, a nonsense mutation was identified. Three of the five genes (*CEP104, CROCC, NEK1*) are members of the ciliary gene family.

**Table 1:** Sanger sequencing results in five tall individuals and two individuals with normal height.

	Mother II.4	Father II.3	Index III.1	Sister III.2	Brother/p II.2	Sister/p II.1	Aunt/p I.3
Height (adjSDS)	0.9	3.2	3.5	2.6	2.9	1.4	2.1
	○	▪	▪	●	▪	○	●
*CEP104 (27)*	w	e	e	e	e	w	e
*CROCC (25)*	w	e	e	e	e	w	e
*NEK1 (25)*	w	e	e	e	e	w	w
*TOM1L2 (26)*	w	e	e	e	e	w	e
*TSTD2 (45)*	w	e	e	e	e	w	w
*AFAP1 (29)*	w	e	e	w	w	w	w
*ANKMY2 (26)*	w	e	e	e	e	e	w
*ARSA (29)*	w	e	e	e	w	e	w
*BBS1 (24)*	w	e	e	e	w	e	w
*BMP2K (27)*	w	e	e	e	w	w	w
*CDC42SE1 (32)*	w	e	e	e	w	e	w
*CHI3L2 (38)*	w	e	e	e	w	e	w
*CHMP6 (23)*	w	e	e	w	w	w	w
*FBXW4 (30)*	w	e	e	w	w	w	w
*FREM1 (33)*	w	e	e	e	w	e	w
*GPATCH2 (39)*	w	e	e	w	w	w	w
*MTX3 (29)*	w	e	e	w	w	w	w
*MYO1C (34)*	w	e	e	w	w	w	w
*MYO18B (26)*	w	e	e	e	w	w	w
*NCOA3 (35)*	w	e	e	w	w	w	w
*NDST1 (26)*	w	e	e	w	e	w	w
*NEDD4L (27)*	w	e	e	w	e	w	w
*RAB27A (29)*	w	e	e	w	w	w	w
*RIN1 (26)*	w	e	e	e	w	e	w
*SLC13A4 (29)*	w	e	e	w	w	w	w
*STAT6 (23)*	w	e	e	w	w	w	w
*STX12 (28)*	w	e	e	e	e	e	e
*TRPC4AP (25)*	w	e	e	w	w	w	w

Twenty-eight genes with high CADD scores above 20 and considered damaging in at least 2/4 in silico prediction tools were analyzed.

Missense variants were found in 24/28 genes: nonsense variants in TSTD2 and CHI3L2; a frameshift in NCOA3 and a splice site variant in FREM1. CADD scores are given in numbers behind the gene symbol; adjSDS, stands for standard deviation score adjusted for secular trend in the population;/p, indicates paternal side; circle, female, square, male; grey color, gene variants shared between the four tall individuals of the index family; orange color, ciliary genes in the five shared candidate genes for tall stature.

We then also had the opportunity to analyze another tall individual, the 94 years old aunt of the father (**Figure 1A**, individual I.3, height SDS 2.1) who showed the identical variants in *CEP104, CROCC*, and *TOM1L2*, indicating that the affected family members in subsequent generations inherited those variants from the proband’s paternal grandmother (I.2), while the *NEK1* and *TSTD2* variants were likely transmitted from the maternal grandfather (I.1) or *de novo* (**Figure 1A**).

### Network Analysis

Network analysis of the five candidate genes for tall stature by the Ingenuity software revealed as “top diseases” *connective tissue and developmental disorders* (1.32 × 10^−3^–2.20 × 10^−4^). Top hit for molecular and cellular function was *cell morphology* (1.49 × 10^−5^–1.49 × 10^−5^). Using the Ingenuity pathway analysis, close network links between the highlighted genes could be revealed. Direct interaction between NEK1 and CEP104 was previously experimentally demonstrated (14, 15). Three of our candidate genes, NEK1, TSTD2, and TOM1L1, directly bind to Exportin 1 (XPO1), a cell cycle-regulated gene which mediates the nuclear transport of cellular proteins to the cytoplasm (16). XPO1 furthermore interacts *via* the Embryonic Ectoderm Development (EED) Polycomb protein with the other candidate gene, CROCC (17) **(** **Figure 2B** **)**. Together, these data highlight experimentally based close network connections between the five genes.

### Expression Analysis in Growth Plates

All candidate genes are expressed ubiquitously according to the genotype-tissue expression GTEx database (www.gtexportal.org). Stature is determined by the growth of long bones, which occurs at the growth plate, a cartilage structure with three principal layers, the resting, proliferative, and hypertrophic zones. To explore possible biological functions of the implicated genes in the growth plate, we assessed mRNA expression of the candidate genes in the growth plates of mice. For this purpose, we evaluated microarray data generated from growth plates of 1-week old mice and RNA-Seq data generated specifically from the proliferative and hypertrophic zones of 1- and 4-week old mouse proximal tibial growth plates (**Supplementary Tables 3A, B** **)**. We found that all five candidate genes are expressed in mouse growth plate. Three of the five genes (*Crocc, Nek1*, and *Tstd2*) showed expression differences between the proliferative and hypertrophic zones with downregulation of *Crocc* and *Nek1* and upregulation of *Tstd2* during hypertrophic differentiation. These differences between proliferative and hypertrophic zones were replicated in a different set of 1-week old mouse growth plate RNA-Seq data containing all three zones (including resting zone) generated independently (unpublished, data not shown). Finally, we looked for changes in gene expression in the mouse growth plate with age, as bone growth slows from 1 to 4 weeks of age, but found little temporal regulation of gene expression with only a decline in expression of *Crocc* in the proliferative zone from 1 to 4 weeks of age.

## Discussion

Our genetic analysis in this family with non-syndromic extreme tall stature argues for an oligogenic origin. To define which of the predicted-deleterious variants might play the strongest role for the phenotype, strict quantitative criteria were applied. Twenty-eight variants were tested and five variants in *CEP104, CROCC, NEK1, TOM1L2*, and *TSTD2* were found to be shared in all tall individuals of the index family. We considered only wildtype variants in the two individuals with normal stature and only heterozygous variants shared between the four tall individuals. Thus, we cannot exclude that other variants from the top list also contribute to the phenotype. Using strict quantitative criteria, we, however, consider it very likely that we have analyzed those genes which play the strongest role for the phenotype. All identified affected amino acids in *CEP104, CROCC, NEK1, TOM1L2*, and *TSTD2* were highly conserved between species down to *Xenopus*, *Fugu* (in the case of CROCC, NEK1, and TOM1L2), *Drosophila* (in the case of NEK1 and TOM1L2) and *C. elegans* (TOM1L2). Three of these five genes (*CEP104, CROCC*, and *NEK1)* are members of the ciliary gene family. Three genes, *CROCC*, *TOM1L2*, and *TSTD2* were found to be associated with height in GWAS studies (www.ebi.ac.uk) (3, 18). Pathway and network analyses furthermore showed close interactions, indicating functionally connected genes.

Longitudinal bone growth occurs at the growth plate by endochondral ossification, where cartilage is formed and then remodeled into bone tissue. This process involves chondrocyte proliferation and hypertrophy and extracellular matrix secretion, orchestrated by complex networks of local paracrine factors and modulated by endocrine factors. All five candidate genes were found to be expressed in mouse growth plate. Expression was also analyzed for temporal and spatial regulation in growth plate cartilage. *Crocc* and *Nek1* were found to be downregulated, and *Tstd2* was found to be upregulated during hypertrophic differentiation, suggesting a possible role of these genes in growth plate chondrocyte differentiation and therefore a possible mechanism to explain the tall stature.

Pathway analysis of the five candidate genes led to connective tissue and developmental disorders as top hits. *NEK1* is the only one of the five genes which has been associated with a phenotype related to height, while *CROCC*, *TOM1L2*, and *TSTD2* were found to be associated with height in GWAS (3, 18). All five genes were shown to be very closely connected in the network analysis, providing evidence for the hypothesis that independent gene variants within connected genes of regulatory pathways can define a certain clinical phenotype (19). Interestingly, the two direct connectors to the candidate genes, *XPO1* and *EED*, themselves present plausible functions related to the phenotype, *XPO1* being a cell cycle-regulated gene and *EED*, mediating suppression of gene activity through histone deacetylation, and being causative for an overgrowth syndrome similar to Weaver syndrome (20, 21).

All five genes are highly promising candidate genes. NEK1 is a serine/threonine-protein kinase, involved in cilium assembly as well as DNA damage checkpoint control and DNA damage repair with relatively high expression in the growth plate. Bi-allelic pathogenic variants, alone or in association with a *DYNC2H1* variant, have previously been associated with syndromic short stature as part of “short-rib thoracic dysplasia 6 with or without polydactyly” (SRTD6, MIM # 263520) (21, 22). Since a *NEK1* variant has been shown to be able to function in concert with other genes in causing a clinical syndrome, we speculate that a combination of the *NEK1* variant and one or more other variants may be the cause of the extreme tall stature in this family.

TOM1L2 has a role in cell cycle progression, intracellular protein transport and regulates growth factor-induced mitogenic signaling. The affected amino acid in TOM1L2 is conserved to worms supporting strong biological significance. Loss of function of the two other ciliary genes (*CEP104* and *CROCC*) or *TSTD2* has not been reported to be associated with a growth disorder, although CROCC has been detected at genome-wide significance for adult height. CROCC (Rooletin) is a structural component of the centrosomes, the main microtubule organizing centre as well as regulator of cell cycle progression. A biallelic defect of *CEP104* causes Joubert syndrome, but no clinical syndrome has been discovered caused by a *CROCC* or *TSD2* variant. However, an interesting observation is that CROCC interacts with RUNX3 (23), while RUNX3 plays an important role in cell cycle regulation and the epiphyseal growth plate. In a recent report, experimental data showed that Runx3 silencing possibly promoted chondrocyte proliferation but suppressed differentiation, which would result in tall stature (24).

Primary cilia are present in almost every cell type including chondrocytes (25). It is remarkable that three of the identified five genes belong to the ciliary gene family. Ciliary genes have been previously connected with several Mendelian causes of multiple growth disorders, probably related to the importance of ciliary-dependent developmental signals, including Hedgehog, Wnt, platelet-derived growth factor, and bone morphogenetic protein (BMP) signaling in the regulation of bone development (26). Almost 20 ciliary genes are associated with skeletal dysplasias (27, 28), and biallelic variants in other ciliary genes (*ALMS1*, *IFT172*, and *KIAA0753*) have been discovered in individuals with growth hormone deficiency (29–31).

Several genes are known in which loss of function is associated with short stature and gain of function with tall stature (2). The best-known example is *SHOX*, of which biallelic defects cause Langer syndrome with severe dwarfism, monoallelic defects Leri–Weill syndrome or non-syndromic short stature, and gene duplications non-syndromic tall stature (32, 33). A similar phenomenon was described for *NPR2* (34). In contrast, for several other genes (e.g., *FGFR3* and *NSD1*) a loss of function is associated with tall stature (CATSHL syndrome and Sotos syndrome, respectively), and gain of function with short stature (*FGFR3* and *CDKN1C*; Achondroplasia and Silver-Russell syndrome) (2). Another gene of which heterozygous loss of function is associated with tall stature and intellectual disability is DNA methyltransferase 3A (*DNMT3A*) (35), while gain of function of DNMT3A leads to dwarfism (36).

In summary, the identified genes in this study encode mechanistically distinct proteins, but their function converges on shared pathways and growth plate-related features. In future studies on genetic causes of stature or skeletal growth, specific attention to these five genes is warranted.

## Data Availability Statement

The datasets presented in this study can be found in online repositories. The names of the repository/repositories and accession number(s) can be found in the article/**Supplementary Material**.

## Ethics Statement

The studies involving human participants were reviewed and approved by Leiden University Medical Center (P06.118) “Genetic diagnosis of very tall stature”. Written informed consent to participate in this study was provided by the participants’ or their legal guardian/next of kin prior to their inclusion in the study. Written informed consent was obtained from the individual(s) and/or minor(s)’ legal guardian/next of kin for the publication of any potentially identifiable images or data included in this article.

## Author Contributions

Conceptualization, JW and GR. Investigation, BW, BE, and JL. Software, NP. Patient resources, CB, HD, and JW. Methodology and supervision, JB and GR. Data analysis, BW, BE, JW, and GR. Writing and editing, JB, JW, and GR. All authors contributed to the article and approved the submitted version.

## Funding

This work was funded by the University of Heidelberg, Medical Faculty.

## Conflict of Interest

The authors declare that the research was conducted in the absence of any commercial or financial relationships that could be construed as a potential conflict of interest.
