# Supplementary material for: Evidence That Non-Syndromic Familial Tall Stature Has an Oligogenic Origin Including Ciliary Genes
Source: Front Endocrinol (Lausanne). 2021 Jun 4;12:660731. doi: 10.3389/fendo.2021.660731 (PMC8237855; doi:10.3389/fendo.2021.660731)
Supplement: Supplementary file 1 [file DataSheet_1.pdf]

Supplementary Information

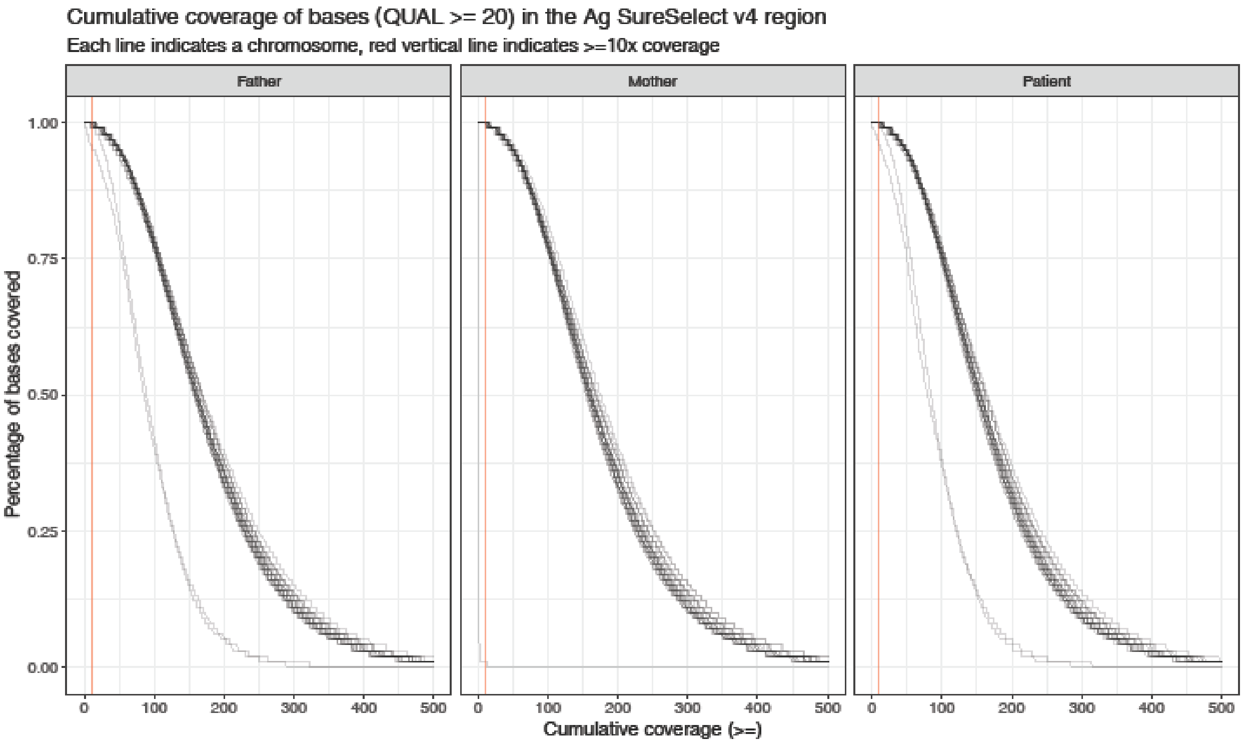

Supplementary Figure 1. Sequence coverage scores

Supplementary Table 1. Primer sequences for amplification by Polymerase chain reaction (PCR)

CEP104	for:	TGGTCCTGATGTCCGAGTGA	rev:	GAGCACAATCCACAGCACT
CROCC	for:	AGCAAGATGAAGGCCAATGAGA	rev:	ACATCACCTCTCAGCCTCCA
NEK1	for:	TGCAGGCATGATATCAGTATTGT	rev:	AAGACTCCTTTATTCCCGCTAAT
TOM1L2	for:	AGTGTCAGCTTATTCGATGCCA	rev:	TTCCCGGGTGTTCTCTTGTG
TSTD2	for:	TGTAGGACAGAGCATAGCGT	rev:	AGCCCCTGACTGATAGCA
AFAP1	for:	TCCAAACCCGGGATCACC	rev:	CTGGGCCTCCTCAGTGT
ANKMY2	for:	ACTCCTGAGCTCCTATGGCT	rev:	GCCCCACCTCCAACATTTG
ARSA	for:	CAAGCACTGCACATACCTGG	rev:	ATGGCGAACTGAGTGACTGA
BBS1	for:	GATTGTAGGACCCAGACGCC	rev:	GGGGAAAGACAGTTGGGGAG
BMP2K	for:	AGGTTTCTGTGGCACA	rev:	CCCGCTGTCACATCATCAGT
CDC42SE1	for:	GGTCTTGAACTCCTGGCCTC	rev:	TGTCAAGGAGATGGCGTCAG
CHI3L2	for:	CTTTCCCCACACTCTGAGCC	rev:	TCGCACTCTCCTCTGCCTAT
CHMP6	for:	CGGCAGGAAGGAGTGAGTG	rev:	AGACCCCTGACCTACCCAC
FBXW4	for:	GAGCTATCACTGCACCCTCC	rev:	CACGTCCCTCTTCCCCTG
FREM1	for:	TGAAGTTCTCAAAACCAGGTCTT	rev:	TAACAATGTGCTGGAGGTGC
GPATCH2	for:	GGTAGGCTCTATCACTGTCCA	rev:	AGTGACTCTGATGACCAAATGT
MTX3	for:	GCCAGCTTACCTCCAAGACT	rev:	CCATGTTGGCCAGGCTTATC
MYO1C	for:	GGGAGGAGGTTACAGTGAGC	rev:	CTCTGACCCTCTTCTGTGGC
MYO18B	for:	CCTAACGGAAACTTTGTGCC	rev:	TCAGGCATATCATTGTTCTCCC
NCOA3	for:	GCATGCCCTTTGTCGCTAAA	rev:	GCCCAATGCCTTCAAACCAA
NDST1	for:	TGCAGGTACATGGAGTTCTTC	rev:	TGAGTGTCAGACGGAGCC
NEDD4L	for:	TGATCAGAAAACAAATGCAAGGT	rev:	ACAAATACGGGTCTTTCAGAAC
RAB27A	for:	ACAGAAGTAGAGCATAAGAGGGCA	rev:	TCACCATGTAGCTTGTTATTGGT
RIN1	for:	CTCTGTCTGGCTGGGGAC	rev:	CCTCAACATCAAGGCTCAGC
SLC13A4	for:	TTAGCATTGACAGGCCAGGG	rev:	TACCCCTCCACATAGTGTCCTGC
STAT6	for:	TCCATACTGAGGCTGTTGTCA	rev:	TTGTGAGCGATATCAGGGGC
STX12	for:	AAGGCTAGTCTCTTACCAAGGT	rev:	AAAATGGAACTGGGACTGCAA
TRPC4AP	for:	AAAGCTCCCGATCACTCAGG	rev:	AGGGGTAGTTTTGCTGGTCT

Supplementary Table 2. Summary of position and type of the identified gene mutations; affected nucleotide and amino acid change; allele number and frequency as well as used prediction tools including CADD, PPH2, Provean, SIFT and Mutation Taster

Gene	Type of mutation	Position	AA change	Nucleotide change	Allele count	Allele number	gnomAD Number of homoz.	Allele frequency	CADD	PPH2	Provean	SIFT	Mutation Taster
CEP104	missense	Chr1:3768920	p.G18S	C>T	22	129134	0	$1.7x10^{-4}$	27	probably damaging	tolerated	neutral	disease causing
CROCC	missense	Chr1:17294861	p.R1675H	G>A	6	66888	0	$0.9x10^{-4}$	25	probably damaging	damaging	deleterious	disease causing
NEK1	missense	Chr4:170523208	p.L55F	C>A	0	0	0	0	25	probably damaging	damaging	deleterious	disease causing
TOM1L2	missense	Chr17:17810800	p.N33T	T>G	2	129166	0	$1.7x10^{-5}$	26	probably damaging	damaging	deleterious	disease causing
TSTD2	stopgain	Chr9:100367147	p.G372*	C>A	90	128534	0	$0.7x10^{-3}$	45	n.a.	n.a.	n.a.	disease causing

Only data of European Non-Finnish individuals was taken into account; AA, amino acids

Supplementary Table 3

A. Supplementary Table 3 A. Expression of candidate genes in proliferative and hypertrophic zones of mouse growth plates at 1 and 4 weeks of age

	Cep104	***Crocc***	***Nek1***	***Tom1l2***	***Tstd2***
1-week Proliferative Zone	410	155	1641	2275	152
1-week Hypertrophic Zone	299	39	344	1914	248
1 week, PZ vs HZ (P-value)	N.S.	0.0019	<0.0001	N.S.	0.029
4-week Proliferative Zone	341	58	1180	2279	144
PZ, 1w vs 4w (P-value)	N.S.	0.011	N.S.	N.S.	N.S.
4-week Hypertrophic Zone	335	51	362	1437	199
HZ, 1w v 4w (P-value)	N.S.	N.S.	N.S.	N.S.	N.S.

RNAseq data are derived from from Lui et al, 2018). PZ, proliferative zone; HZ, hypertrophic zone; N.S., not significant (comparative data between proliferative and hypertrophic zone)

B. Expression of candidate genes in mouse growth plate versus soft tissues

Gene Symbol	*Crocc*	*Nek1*	*Tom1l2*	*Tstd2*
RefSeq Transcript ID	NM_001145958<br>NM_172122	NM_175089	NM_001039092<br>NM_001039093<br>NM_153080	NM_173033
**Growth plate vs heart**				
fold-difference	1.14	5.05	-1.94	1.90
p-value	0.25	<0.0001	<0.0001	<0.0001
**Growth plate vs kidney**				
fold-difference	-1.53	4.09	-1.66	2.10
p-value	0.0011	<0.0001	0.00074	<0.0001
**Growth plate vs lung**				
fold-difference	1.16	4.70	-1.23	2.12
p-value	0.20	<0.0001	0.11	<0.0001

Microarray data are given as fold change from Lui et al, 2012. Positive fold differences indicate greater expression in growth plate than in the comparator tissue; Negative fold differences indicate greater expression in the comparator tissue than in growth plate.

References

Lui JC, Nilsson O, Chan Y, Palmer CD, Andrade AC, Hirschhorn JN and Baron J. Synthesizing genome-wide association studies and expression microarray reveals novel genes that act in the human growth plate to modulate height. *Hum Mol Genet*. 2012; 21:5193-201.

Lui JC, Jee YH, Garrison P, Iben JR, Yue S, Ad M, Nguyen Q, Kikani B, Wakabayashi Y, **Baron J** Differential aging of growth plate cartilage underlies differences in bone length and thus helps determine skeletal proportions. *PLoS Biol.* 2018 Jul 23;16(7):e2005263. doi: 10.1371/journal.pbio.2005263.
